# Mechanistic Insight into Intestinal α-Synuclein Aggregation in Parkinson’s Disease Using a Laser-Printed Electrochemical Sensor

**DOI:** 10.1021/acschemneuro.4c00106

**Published:** 2024-07-03

**Authors:** Julia M. Balsamo, Keren Zhou, Vinay Kammarchedu, Aida Ebrahimi, Elizabeth N. Bess

**Affiliations:** † Department of Chemistry, University of California, Irvine, California 92617, United States; ‡ School of Electrical Engineering and Computer Science, The Pennsylvania State University, University Park, Pennsylvania 16802, United States; § Materials Research Institute, The Pennsylvania State University, University Park, Pennsylvania 16802, United States; ∥ Department of Biomedical Engineering, The Pennsylvania State University, University Park, Pennsylvania 16802, United States; ⊥ Department of Molecular Biology and Biochemistry, University of California, Irvine, California 92617, United States

**Keywords:** α-synuclein, Parkinson’s disease, dopamine, iron, polyphenols, LIG sensor

## Abstract

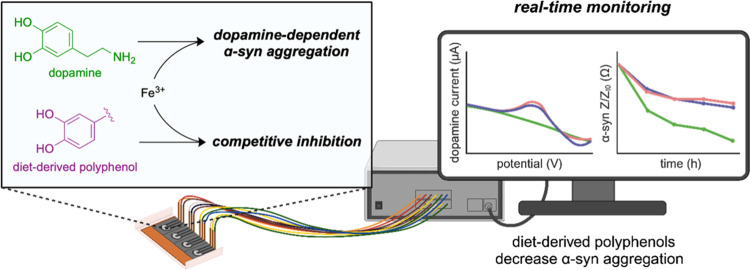

Aggregated deposits of the protein α-synuclein and depleting levels of dopamine in the brain correlate with Parkinson’s disease development. Treatments often focus on replenishing dopamine in the brain; however, the brain might not be the only site requiring attention. Aggregates of α-synuclein appear to accumulate in the gut years prior to the onset of any motor symptoms. Enteroendocrine cells (specialized gut epithelial cells) may be the source of intestinal α-synuclein, as they natively express this protein. Enteroendocrine cells are constantly exposed to gut bacteria and their metabolites because they border the gut lumen. These cells also express the dopamine metabolic pathway and form synapses with vagal neurons, which innervate the gut and brain. Through this connection, Parkinson’s disease pathology may originate in the gut and spread to the brain over time. Effective therapeutics to prevent this disease progression are lacking due to a limited understanding of the mechanisms by which α-synuclein aggregation occurs in the gut. We previously proposed a gut bacterial metabolic pathway responsible for the initiation of α-synuclein aggregation that is dependent on the oxidation of dopamine. Here, we develop a new tool, a laser-induced graphene-based electrochemical sensor chip, to track α-synuclein aggregation and dopamine level over time. Using these sensor chips, we evaluated diet-derived catechols dihydrocaffeic acid and caffeic acid as potential inhibitors of α-synuclein aggregation. Our results suggest that these molecules inhibit dopamine oxidation. We also found that these dietary catechols inhibit α-synuclein aggregation in STC-1 enteroendocrine cells. These findings are critical next steps to reveal new avenues for targeted therapeutics to treat Parkinson’s disease, specifically in the context of functional foods that may be used to reshape the gut environment.

## Introduction

The aggregation of the protein α-synuclein (α-syn) in dopaminergic neurons has been linked to Parkinson’s disease (PD) development.^1^ While PD etiology is generally associated with the brain, mounting evidence supports a hypothesis proposed by Braak et al.: some PD subtypes may originate in the gastrointestinal (GI) tract.^2,3^ Enteroendocrine cells (EECs) located in the intestinal wall express α-syn, and aggregates of α-syn accumulate in the gut at least eight years prior to the onset of motor symptoms in patients with idiopathic PD.^4,5^ These cells additionally express the dopamine (DA) metabolic pathway.^6^ EECs border the gut lumen; as such, they are exposed to the gut microbiota and its metabolites.^4^ Interestingly, EECs connect to the enteric nervous system, which synapses with the central nervous system via the vagus nerve. It was recently demonstrated that EECs can propagate α-syn aggregates to neighboring neuronal cells through cell–cell contact.^7^ It has also been shown that aggregates of α-syn injected into the intestinal wall of rodents were transported to the brain via the vagus nerve.^8,9^ Taken together, these findings suggest that PD pathology may originate in the gut and spread to the brain.^3,10^

**Scheme 1:**
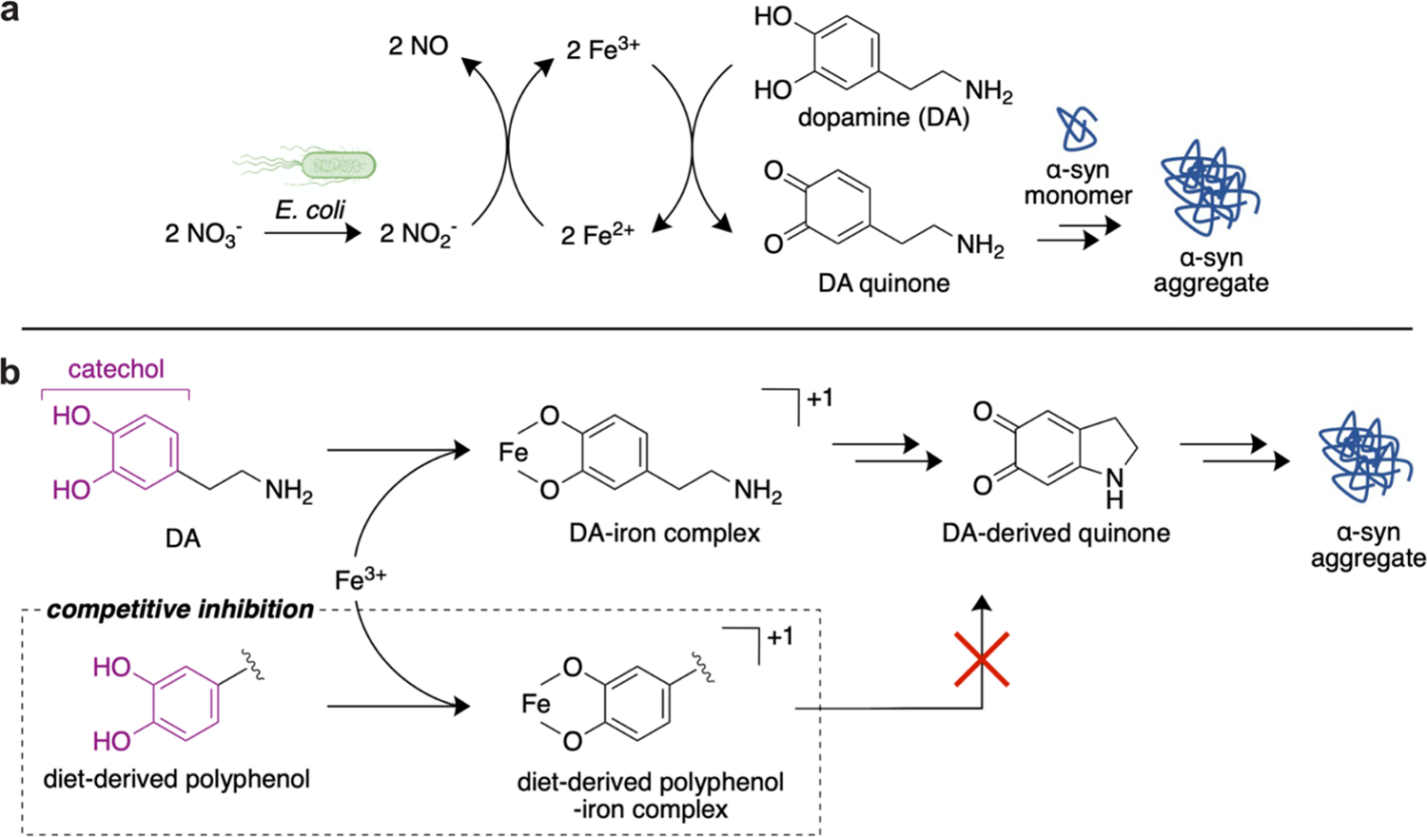
Proposed Mechanisms for (a) the Aggregation of α-Syn in the GI Tract and (b) Competitive Inhibition of α-Syn Aggregation in the Presence of Diet-Derived Catechols

In our previous work, we reported a molecular-level mechanism by which gut bacteria mediate the aggregation of α-syn.^11^ Central to this mechanism is the *Enterobacteriaceae* family of facultative anaerobic bacteria, which is often enriched in the gut microbiotas of people with PD.^12,13^ We demonstrated that when these bacteria perform nitrate respiration, a metabolic product—nitrite—initiates a cascade of oxidation reactions, resulting in the aggregation of α-syn (Scheme 1a). Our proposed mechanism parallels suggested pathways in the brain involving iron-mediated DA oxidation;^14−16^ similar to the brain, in intestinal epithelial cells, there is also a convergence of concentrated iron^17^ and DA (46% of the body’s DA pool is contained in the gastrointestinal tract).^18−20^ We determined that nitrite oxidizes Fe^2+^ to Fe^3+^, which then interacts with the catechol moiety in DA to form DA-derived quinones. When DA-derived quinones interact with the Y_125_EMPS_129_ pentapeptide sequence in α-syn, the protein misfolds and subsequently aggregates.^21^ We found that DA oxidation is crucial to nitrite-induced α-syn aggregation. As such, strategies that prevent DA oxidation are expected to limit aggregate formation.

Diet may be a source of molecules that inhibit intestinal DA oxidation and α-syn aggregation. Because gut microbiome composition and function are shaped by diet, functional foods that alter gut microbial chemistry implicated in PD onset could be used to prevent this disease.^22^ Specifically, compounds with catechol moieties could limit α-syn aggregation by outcompeting catechol in DA for binding Fe^3+^ (Scheme 1b). Polyphenols—abundant in vegetables, fruits, nuts, and whole grains—often contain at least one catechol moiety. Curiously, a Mediterranean diet is rich in polyphenols and is associated with reductions in PD risk and severity as well as delayed disease onset.^23−25^

**Figure 1:**
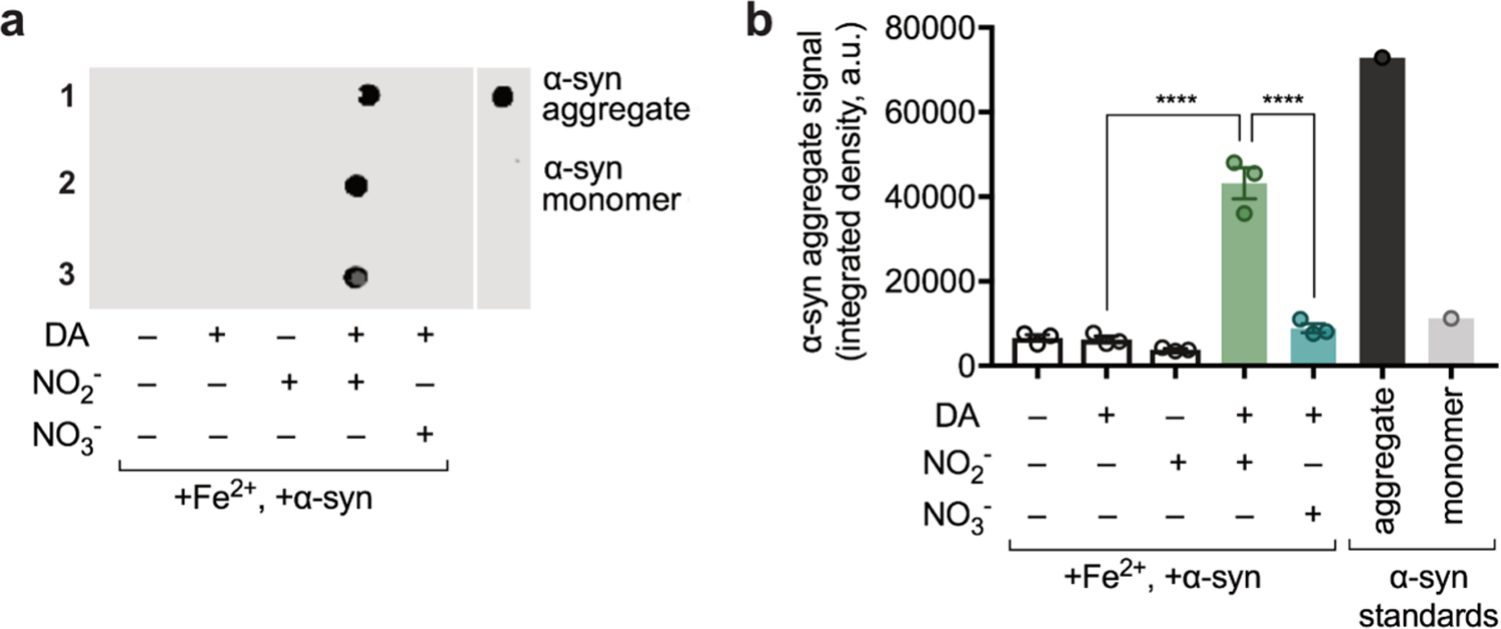
Assessment of α-syn aggregation using dot blot. (a) Dot blot spotted with biological replicates 1–3 after a 4 h incubation at 37 °C under anoxic conditions and immunostained with anti-α-syn aggregate primary antibody MJFR-14. (b) Dot blot quantification of α-syn aggregation (all error bars represent S.E.M. for *n* = 3 samples; significance determined by one-way ANOVA with Sidak’s multiple comparison test, ****: *P* < 0.0001).

We sought to identify diet-derived catechol inhibitors of α-syn aggregation to provide new insights into potential therapeutics to treat and prevent PD. However, we were limited in our ability to screen and track this aggregation process—and subsequently its inhibition—in real time. In our previous work, we performed immunostaining of dot blots to quantify the end-point extent of α-syn aggregation (Figure 1). However, this method, as well as other commonly used methods (e.g., enzyme-linked immunosorbent assay, ELISA^26^), limits the ability to analyze the oxidation of DA and subsequently the aggregation of α-syn in real time, occluding a window onto the dynamics of this process.

Electrochemical techniques offer simpler, portable, and real-time monitoring compared to ELISA, conventional circular dichroism spectroscopy,^27^ and microscopy^28^ methods used to measure α-syn aggregates. Currently, several electrochemical sensors have been developed to detect α-syn; however, they usually require antibodies to capture molecules, which limit their use for real-time studies. For example, Bryan et al. developed impedance sensors based on polyethylene glycol–gold electrodes functionalized with α-syn antibody.^29^ Undiluted blood serum from patients was used to benchmark the sensors. Charge transfer resistance of samples from PD patients was 7-fold higher compared to the control. In another work, Sun et al. reported a highly selective aptasensor to specifically detect the α-syn oligomer via impedance, surface plasmon resonance, and colorimetry methods.^30^ The results showed a significant increase in the charge transfer resistance after the capture of α-syn oligomers on the aptamer-covered electrode surface. These sensors require that costly biomolecules (antibodies or aptamers) coat the electrodes to specifically capture α-syn for electrochemical measurements. Developing a simpler electrode that does not require such specialized coatings would facilitate sensor fabrication, thereby expanding their utility.

In addition to monitoring α-syn aggregation, simultaneously examining the role of DA oxidation offers a more precise means of understanding the mechanisms controlling α-syn aggregation. Given the redox activity of DA and unique advantages of electrochemical devices for profiling redox reactions, several electrochemical sensors have been developed for detection and monitoring of DA, from *in vitro* assays^31−33^ to *in vivo* probes.^34−36^ In the building of DA sensors, various electrode materials have been explored, including graphene, ionic liquids, and nanoparticles. Among them, graphene-based electrochemical sensors have attracted special attention due to the favorable aromatic π–π stacking and electrostatic attraction between positively charged DA and the negatively charged graphene surface.^37^ Different forms of graphene and its derivatives have been utilized in manufacturing DA sensors using a variety of techniques, including drop-casting,^38^ inkjet/aerosol jet printing,^39^ screen-printing,^40^ lithography,^41^ and direct laser writing.^42^ For example, Butler et al. reported a facile and scalable way to fabricate screen-printed sensors using graphene ink to detect DA as low as 5 pM in serum using differential pulse voltammetry.

An alternative strategy employs laser-induced graphene (LIG), which is graphene with a three-dimensional (3D) porous network derived from commercial polymer films by using a low-cost/maskless direct writing process.^43^ LIG holds great promise as an ideal material for biosensor development, as it is easy to pattern and fabricate; it is also cost-effective and environmentally friendly compared to other methods. Several DA biosensors based on LIG have been reported. For example, Hui et al. developed a highly flexible and selective electrochemical sensor with a Pt–Au nanoparticle-modified LIG electrode for the detection of dopamine.^44^ The electrodeposited Pt–Au nanoparticles increased the surface area of the electrode and improved sensitivity and selectivity of the sensor.

While multiple reports have used electrochemical sensors for DA detection, there are no reports of real-time electrochemical monitoring of depleting levels of DA in the context of α-syn aggregation related to PD. The real-time, label-free, and simultaneous probing of these molecules is expected to offer a better understanding of α-syn aggregation mediated by gut microbiota. In this work, we developed LIG-based electrochemical sensor chips that simultaneously track, in real time, changes in DA levels (using square wave voltammetry) and aggregation of α-syn (using impedance monitoring). This enabled our investigation of potential inhibitors of both oxidation of DA by Fe^3+^ and aggregation of α-syn. Specifically, we tested the diet-derived catechols dihydrocaffeic acid (DHCA) and caffeic acid (CA). As demonstrated by our experiments using the developed portable LIG-based electrochemical sensors and immunofluorescence microscopy assays in model EECs, the presence of these dietary catechols significantly limits the extent of α-syn aggregation, likely as a result of the lower levels of DA-derived quinones forming. Ultimately, the developed sensor chips open new avenues for rapid screening and discovery of small molecules to prevent DA-dependent α-syn aggregation.

## Results

### Sensor Fabrication, Optimization, and Calibration

**Figure 2:**
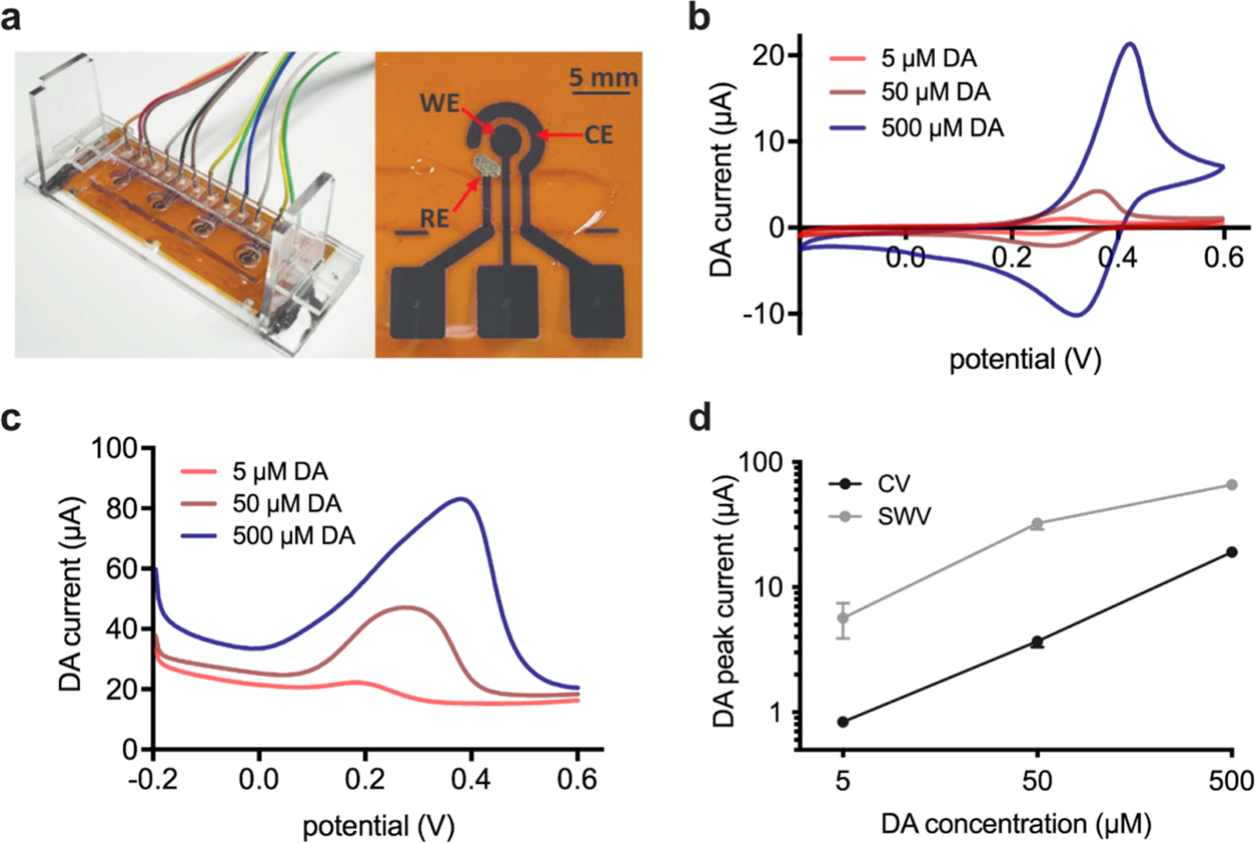
Calibrating electrochemical measurement of DA in ambient air. (a) Optical images of a single electrochemical sensor module (left) and enlarged view of a single 3-electrode sensor (right). (b) Representative CV curve of DA current at various concentrations (5, 50, and 500 μM). (c) Representative SWV curve of DA current at various concentrations (5, 50, and 500 μM). (d) Calibration curves based on CV and SWV DA peak current data.

The electrochemical sensor chips were prepared based on LIG with a three-electrode configuration. The LIG pattern was fabricated using a commercial CO_2_ laser engraving machine, as previously reported.^45^ Briefly, a polyimide sheet is placed in a laser engraving setup, and the graphene electrodes are patterned by laser irradiation. Herein, we designed a test module containing four individual sensors. To hold the test solution, a small PDMS chamber (8 mm diameter and 5 mm height for each sensor) was made by casting and curing PDMS in a 3D-printed mold. The PDMS chamber and the sensors were bonded together using ecoflex silicone. Before electrochemical testing, the sensor was mounted in a homemade holder made with an acrylic board with wire connections. Figure 2a shows an as-prepared individual sensor and a sensor array module with the connections.

In electrochemical sensors, the reference electrode (RE) plays a key role in achieving a stable potential on the working electrode (WE). In order to fabricate a stable on-chip pseudoreference electrode (pRE), we first studied two different approaches to functionalize LIG with silver (Ag). The first method used the direct laser writing of Ag by the second lasing of a 5 μL droplet of AgNO_3_ solution on LIG as the RE. Different concentrations of AgNO_3_ were studied. As illustrated in Figure S1a, the potential between the RE and the WE in PBS is not stable. As a result, the measurement of the oxidation peak of DA shifts over time (Figure S1b). In the second approach, we employed electrodeposited Ag using a previously reported process.^45^ Electrodeposited Ag pRE yielded a stable potential between the RE and WE; hence, there was no significant shift of the DA peak. Consequently, the sensors for all of the following results were fabricated by using electrodeposited Ag pRE.

To determine the peak profile in response to DA and prepare the calibration curves, the sensors were first tested with DA hydrochloride (DA; 5, 50, or 500 μM) in 50 mM sodium nitrate (NO_3_^–^) in an ambient laboratory environment. We implemented two different voltammetry methods, including cyclic voltammetry (CV) and square wave voltammetry (SWV). Figure 2b,c shows representative CV and SWV curves, respectively, and Figure 2d depicts the calculated calibration curves for each. While the calibrated CV curve exhibits a slightly better linear response, SWV offers a higher current value and so is less prone to noise. As such, for analytical purposes in the experiments with α-syn, we used the SWV signals.

We also sought to determine whether the DA calibration curves prepared from samples under ambient laboratory conditions are suitable to be used for anaerobic analyses. We accomplished this by performing the voltammetry tests in a custom-made anaerobic glovebox (Figure S2) purged with 20% CO_2_ and 80% N_2_. This glovebox was also equipped with the necessary circuit modules to perform multiplexed electrochemical testing. As shown in Figure S3, no significant difference in the sensitivity to DA (SWV current peak height) was found between glovebox tests and the ambient atmosphere.

### Simultaneous Monitoring of DA Oxidation and α-Syn Aggregation using LIG Sensors

**Figure 3:**
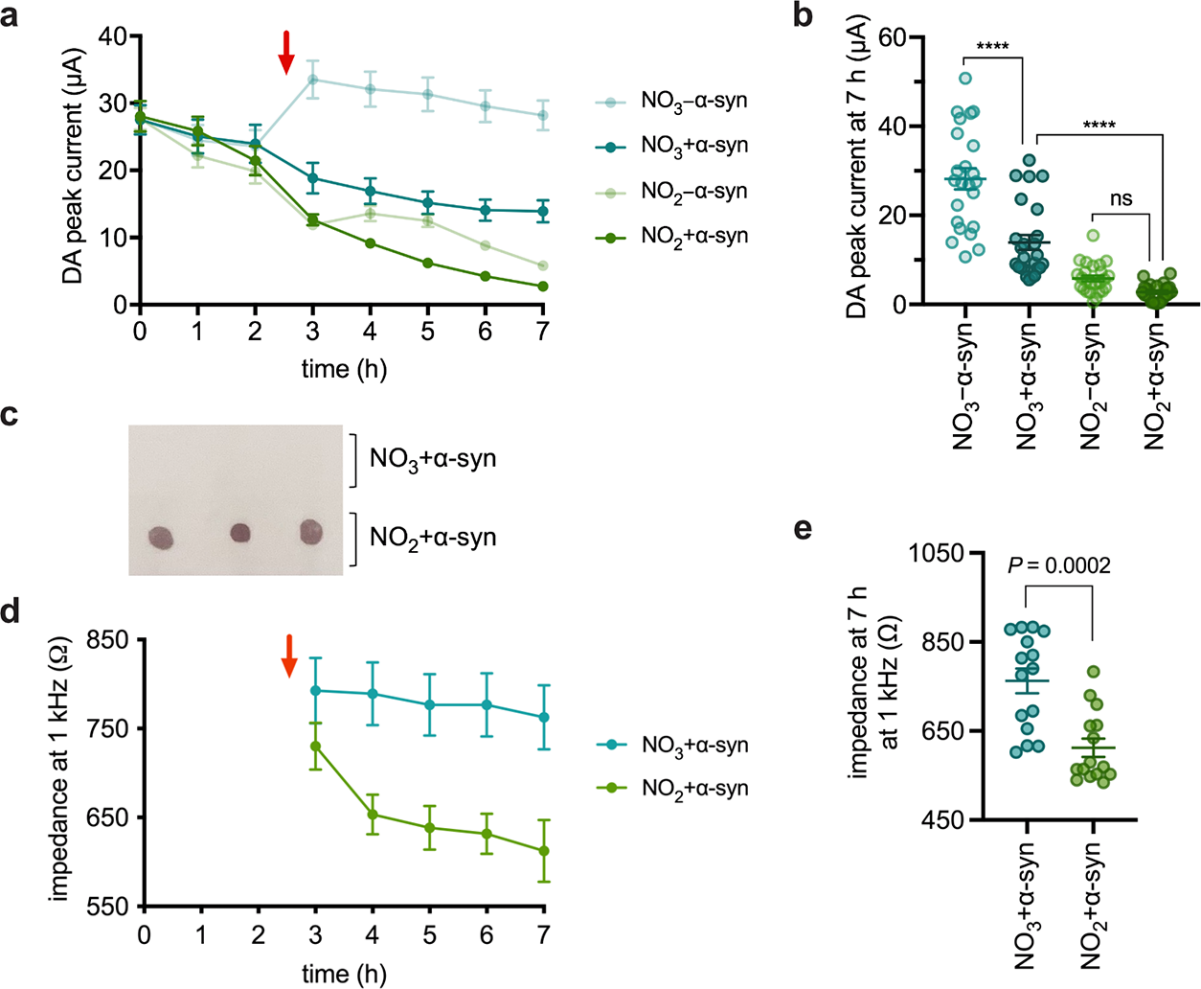
Electrochemical measurement of DA and α-syn aggregation. (a) SWV DA peak current for samples NO_3_–α-syn, NO_3_ + α-syn, NO_2_–α-syn, and NO_2_ + α-syn. Red arrow denotes the time at which α-syn and NO_3_^–^ or NO_2_^–^ were added. (b) SWV DA peak current at 7 h for each sample in (a) (error bars represent S.E.M.; *n* = 20 biological replicates per condition; significance determined by one-way ANOVA with Sidak’s multiple comparison test, ****: *P* < 0.0001, ns: not significant) (c) Dot blot stained with nitroblue tetrazolium (NBT) to detect the formation of quinones across three biological replicates. (d) Impedance measurements at 1 kHz for NO_3_ + α-syn and NO_2_ + α-syn following the addition of α-syn. Red arrow denotes the time at which α-syn and NO_3_^–^ or NO_2_^–^ was added. (e) Impedance at 7 h for each sample in panel (d) (error bars represent S.E.M.; *n* = 20 biological replicates per condition).

As previously reported, nitrite generated during *Enterobacteriaceae* nitrate dissimilatory metabolism oxidizes Fe^2+^ to Fe^3+^.^11^ Fe^3+^ can then oxidize DA, which leads to α-syn aggregation. Before voltammetry measurements were performed, the glovebox was purged with a mixture of 20% CO_2_ and 80% N_2_ to obtain an O_2_ level lower than 0.5%. This anaerobic chamber’s temperature was set to 37 °C. Chemicals and as-printed sensors were transferred into the chamber for the experiments. We prepared four test groups for monitoring DA levels and α-syn aggregation. Each solution contained Fe^2+^ (1.33 mM) and DA (1.33 mM) and was allowed to equilibrate for 2 h in order for the DA peak current to stabilize. After 3 h (indicated by the red arrow in Figure 3a,d), the solutions were appended with NaNO_3_ or NaNO_2_ and either with α-syn monomer (NO_3_ + α-syn; NO_2_ + α-syn) or without (NO_3_–α-syn; NO_2_–α-syn). The SWV peak current was measured as a function of the DA level over 7 h. As shown in Figure 3a, samples containing NO_2_^–^ resulted in a lower DA peak current in comparison to those containing NO_3_^–^. For instance, at 7 h, the SWV DA peak current was 5.1-fold higher for NO_3_ + α-syn compared to NO_2_ + α-syn (Figure 3b). These data suggest that DA was depleted in the presence of NO_2_^–^. Nitroblue tetrazolium (NBT) staining of dot blots spotted with similarly prepared solutions enabled the detection of any quinones present.^46^ We observed a higher level of quinones in NO_2_ + α-syn as compared to NO_3_ + α-syn, indicating that depleted DA levels monitored with our sensor correlate to elevated levels of oxidation and quinone formation (Figure 3c). To confirm that the reduced DA peak is not due to the electrode fouling after a long time measurement, we immersed LIG sensors in 500 μM DA with 50 mM NaNO_3_ for 7 h. The sensors were tested with fresh DA solutions at the beginning and end of the immersion. As Figure S4 illustrates, the sensitivity of the LIG sensor remains almost the same after 7 h, confirming stability of the sensors for continuous, long-term monitoring. Of note, similar sensors (i.e., Nafion-covered LIG) have been previously used for real-time monitoring of bacterial biofilms over several days, demonstrating their suitability for continuous analysis in complex media.^45,47^

We previously reported that α-syn forms aggregates in the presence of DA and NO_2_^–^ but not NO_3_^–^.^11^ Here, we observed that NO_2_ + α-syn (in which the DA level is the lowest putatively due to DA oxidation) afforded an impedance at 1 kHz that was much lower than that of NO_3_ + α-syn (which exhibited a higher DA level indicative of less DA oxidation), as shown in Figure 3d. Strikingly, after only ∼4 h following the addition of α-syn (*t* = 7 h), NO_3_ + α-syn afforded a 1.3-fold higher impedance than did NO_2_ + α-syn (Figure 3e). A similar trend was observed when the impedance measured in a sample of a commercially prepared α-syn aggregate was compared to the impedance of the α-syn monomer control (Figure S5). The different impedance of α-syn monomers and aggregates indicates that impedance measurement can be used to track the formation of α-syn aggregates over time.

### Monitoring Competitive Inhibition of α-Syn Aggregation by Dietary Catechols

**Figure 4:**
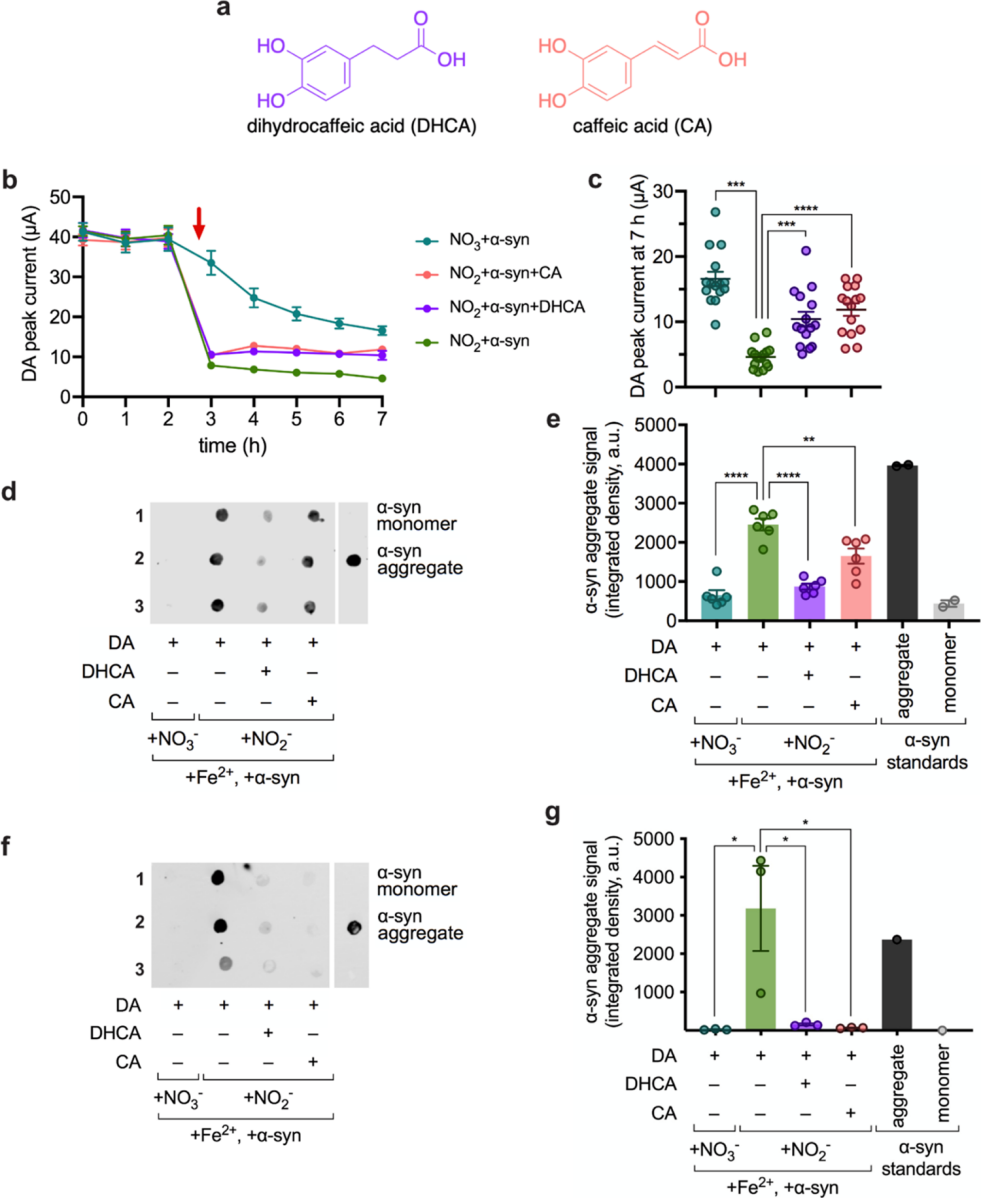
Screening potential inhibitors of dopamine oxidation and α-syn aggregation. (a) Structures of the screened diet-derived catechols dihydrocaffeic acid (DHCA) and caffeic acid (CA). (b) DA peak current for NO_3_ + α-syn, NO_2_ + α-syn, NO_2_ + α-syn + DHCA, and NO_2_ + α-syn + CA. Red arrow denotes the time at which α-syn and NO_3_^–^ or NO_2_^–^ was added and (c) depicts the DA peak current at 7 h for each sample. (d) Representative dot blot spotted with biological replicates 1–3 and immunostained with anti-α-syn aggregate primary antibody MJFR-14. (e) Respective quantified aggregate signal across two dot blots probed with MJFR-14, *n* = 3 biological replicates. (f) Representative dot blot spotted with biological replicates 1–3 and immunostained with anti-α-syn aggregate primary antibody 5G4. (g) Respective quantified aggregate signal across the dot blot in panel (f) probed with 5G4, *n* = 3 biological replicates (all error bars represent S.E.M.; significance determined by one-way ANOVA with Sidak’s multiple comparison test, ****: *P* < 0.0001,***: *P* = 0.002 for the DA peak current at 7 h, **: *P* = 0.0012, *: *P* = 0.0115, 0.0145, and 0.0124 from left to right for the quantified dot blots).

After establishing that the LIG-based sensors enable the monitoring of DA-dependent α-syn aggregation, we sought to implement this tool to identify potential inhibitors of this process. As diet may be a source of molecules that inhibit intestinal DA oxidation and α-syn aggregation, we screened CA due to its high abundance in the diet,^48^ particularly in coffee and fruit. DHCA is a bacterial metabolite of CA;^49^ thus, we selected it to determine whether any inhibitory activity is lost or, conversely, is more potent when CA is metabolized. We hypothesized that these diet-derived polyphenols (Figure 4a) could limit DA-dependent α-syn aggregation by outcompeting the catechol in DA for iron binding, thereby limiting DA oxidation (Scheme 1b). To test the inhibitory effects of DHCA and CA, each was incubated with DA, the α-syn monomer, Fe^2+^, and NO_2_^–^ (NO_2_ + α-syn + DHCA and NO_2_ + α-syn + CA, respectively). Samples of NO_3_ + α-syn and NO_2_ + α-syn furnished negative and positive controls of α-syn aggregation, respectively. Upon measuring the DA peak current, we observed that both NO_2_ + α-syn + DHCA and NO_2_ + α-syn + CA displayed higher DA levels than did NO_2_ + α-syn (which contains DA but no DHCA or CA). The real-time sensor data suggest that DHCA and CA limited oxidation of DA (Figure 4b). At 7 h, NO_2_ + α-syn + DHCA and NO_2_ + α-syn + CA retained 2.3-fold and 2.6-fold higher DA levels, respectively, than the DA level in NO_2_ + α-syn (Figure 4c). DHCA and CA could be sequestering the Fe^3+^,^50^ limiting Fe^3+^-mediated DA oxidation and, thus, maintaining higher DA levels. Because DA oxidation is crucial for α-syn aggregation, our findings position DHCA and CA as candidate inhibitors of nitrite-induced DA-dependent α-syn aggregation.

Thus, we next tested the impact of DHCA and CA on α-syn aggregation. By immunostaining dot blots with anti-α-syn aggregate antibodies, we quantified the amount of aggregate formation in each sample after a 4 h incubation at 37 °C under anoxic conditions (Figure 4d,f). Across all blots, significant α-syn aggregation was induced by NO_2_ + α-syn compared to that by NO_3_ + α-syn, as expected. Excitingly, appending NO_2_ + α-syn with candidate inhibitors DHCA (NO_2_ + α-syn + DHCA) or CA (NO_2_ + α-syn + CA) inhibited the formation of both α-syn fibrils (detected by the MJFR-14 antibody; Figure 4d–e) and the more pathogenic oligomers (detected by the 5G4 antibody; Figure 4f–g).^51^ The ability of DHCA and CA to limit α-syn aggregation is in alignment with the higher levels of DA measured in these samples as compared with NO_2_ + α-syn at 7 h (Figure 4b). Moreover, the inhibition of oligomer formation by DHCA and CA was similar to that observed when using iron-chelating EDTA,^52^ which putatively limits iron oxidation, and ATP, which limits Fe^3+^-catalyzed DA oxidation (Figure S6).^53^ Taken together, our sensor enables the real-time screening of promising inhibitors of DA-dependent α-syn aggregation.

### Diet-Derived Catechols Mitigate α-Syn Aggregation in Gut Epithelial Cells

We next sought to determine whether the diet-derived catechols that limited the extent of α-syn aggregation *in vitro* would show promise in the mammalian gut. Murine STC-1 cells (an accepted model cell line for EECs)^54^ were used to investigate the potential of these catechols to inhibit α-syn aggregation. Because EECs naturally express α-syn^4^ as well as the DA metabolic pathway,^6^ only NO_3_^–^ (50 mM) or NO_2_^–^ (50 mM), as well as varying concentrations of DHCA or CA (25, 50, and 100 μM), were added to STC-1 cells to test their impacts on α-syn aggregation *in cellulo*.

**Figure 5:**
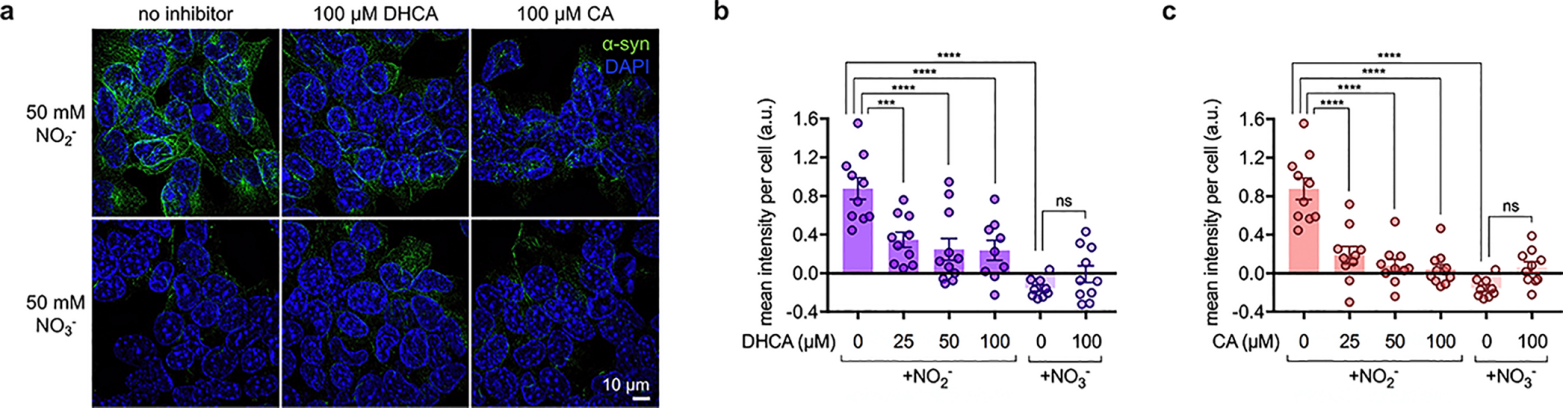
Nitrite-induced aggregation of α-syn in gut epithelial cells is reduced in the presence of diet-derived catechols DHCA and CA. (a) Representative images of fixed STC-1 cells incubated for 24 h with NO_2_^–^ or NO_3_^–^ and DHCA or CA. α-Syn aggregate signal is in green, and DAPI-stained nuclei are in blue. Mean fluorescence intensity per cell was quantified from maximum intensity projections acquired by structured illumination microscopy for (b) DHCA and (c) CA (*n* = 3 independent biological replicates, 3–4 technical replicates for each, bars denote mean ± S.E.M.; significance determined by one-way ANOVA with Sidak’s multiple comparison test, ****: *P* < 0.0001, ***: *P* = 0.0009).

Intracellular α-syn aggregates were quantified by immunofluorescence microscopy. Fixed STC-1 cells were probed using an antibody with an affinity for α-syn aggregates (Figures 5a and S7a). Across three independent replicates, significant α-syn aggregation was induced in the presence of NO_2_^–^ but not in the presence of NO_3_^–^, as compared with the untreated controls. Supplying NO_2_^–^-treated cells with DHCA or CA attenuated α-syn aggregation relative to samples without an inhibitor, with 2.5-fold and 4.6-fold less aggregation observed with as little as 25 μM DHCA or CA added, respectively (Figure 5b,c). Isotype controls indicate no significant nonspecific binding (Figure S7b). Treatments with higher concentrations of either DHCA or CA further reduced the level of α-syn aggregation. Cells treated with 50 μM DHCA resulted in a 3.6-fold decrease in aggregation; 100 μM DHCA resulted in a 3.7-fold decrease. Astoundingly, cells treated with 50 or 100 μM CA resulted in 10.9-fold or 58.2-fold decreases in aggregation, respectively. These findings not only provide strong evidence of a diet-based strategy to inhibit α-syn aggregation in the gut but also support the functionality of our sensors for identifying such promising inhibitors.

## Discussion

In this work, we developed a low-cost, portable sensor array for simultaneous probing and tracking of changes in DA levels and the aggregation of α-syn over time. The electrochemical sensor chips—fabricated using laser-induced graphene—monitor in real time the depleting levels of DA by measuring its oxidation peak current using SWV as well as simultaneous impedance measurements to signal α-syn aggregation. Findings from NBT staining to detect quinones suggested that the depleting levels of DA detected by the sensor correlated with high levels of DA quinone formation, which is necessary for α-syn aggregation. By measuring real-time electrochemical signals confirmed with an end-point dot blot assay, we were able to screen diet-derived catechols DHCA and CA as potential inhibitors of DA-oxidation-mediated α-syn aggregation. The sensor chips enable the tracking of aggregate formation in the presence of various inhibitors of DA oxidation in real time over 4 h. Both real-time electrochemical signals and immunostained dot blot end-point assays suggest that the diet-derived catechols DHCA and CA competitively inhibit the oxidation of DA by Fe^3+^, limiting the amount of α-syn aggregates formed. Our findings using model EECs further support the biological relevance of these promising diet-derived inhibitors to the mammalian gut. Aggregation of endogenous α-syn was significantly limited in the presence of either DHCA or CA, despite the addition of high concentrations of NO_2_^–^. Taken together, these results demonstrate proof-of-concept for rapid screening of various potential inhibitors using the developed low-cost sensors, which only require a few microliters of sample. Continued development and application of this method is expected to enable the discovery of additional candidate inhibitors to prevent intestinal α-syn aggregation and Parkinson’s disease.

## Materials and Methods

### Sensor Fabrication

The LIG 3-electrode pattern with a center working electrode (WE, 2 mm diameter) surrounded by the concentric counter (CE) and reference electrode (RE) was designed in AutoCAD and then manufactured by a laser engraving machine (VSL2.30, Universal Laser Systems) using the processing parameter we previously reported,^45^ i.e., power: 10.5%, speed: 5.5%, points per inch: 1000. All printing steps were done with a pulse per inch (PPI) count of 1000 in raster mode. Then, RE was deposited with silver as the pseudoreference electrode (pRE). Two methods were investigated to identify the most stable RE. To improve the stability and selectivity of the sensors, 2 μL of 5% Nafion dispersion (D-521, Thermo Scientific Chemicals) was dropped on the WE surface. The test chamber (diameter = 8 mm) was made by casting poly(dimethylsiloxane) (PDMS, Dow Sylgard 184) into a 3D-printed mold. Finally, the printed electrodes and PDMS were bonded using Ecoflex silicone.

### Electrochemical Measurements

Electrochemical characterization was performed using a MultiPalmSens4 multichannel potentiostat (from PalmSens; capable of measuring 4 channels in parallel) with a custom-made multiplexer circuit, capable of multiplexing 20 sensor chips. The custom multiplexer consists of commercial electromagnetic relays connecting the sensor electrodes to a potentiostat channel. The multiplexer was interfaced to the potentiostat by using auxiliary digital control pins. The instrument and the custom multiplexer were controlled with MultiTrace 4.5 software. For the electrochemical measurements, the chemicals and the as-printed sensor array were transferred to the glovebox, followed by mixing the reagents and application to the sensor wells. Then, the sensor modules were connected to the electrochemical interface using a spring connector with holders. DA and FeCl_2_ were mixed with nitrate or nitrite and first added to the PDMS chamber of each sensor 3 h before the aggregation test to pretreat the surface of the sensor. This initial pretreatment ensured that the highly porous LIG electrodes were properly wetted by the liquid before the addition of the key reagents (nitrate or nitrite, monomeric α-syn, and catechols). At ∼2.8 h, the rest of the chemicals were added to the chamber and mixed by gentle pipetting using a micropipette. The electrochemical measurement started immediately after the chemicals were mixed, and each measurement took ∼12 min. Then, the sensor was left for continuous measurement over another 4 h (total measurement time of 7 h). Four sensors on the same chip (see Figure 2a) were measured at once, after which the multiplexer switched the connection to the next sensor chip. Cyclic voltammetry (CV) measurements were performed with the following parameters: scan rate = 50 mV/s, *E*_step_ = 5 mV, potential range = −0.2 to 0.8 V, and number of scans = 3. Square wave voltammetry (SWV) measurements were performed with the following parameters: frequency = 15 Hz, *E*_pulse_ = 50 mV, *E*_step_ = 5 mV, potential range = −0.2 to 0.8 V. Electrochemical impedance spectroscopy (EIS) measurements were performed with the following parameters: Vac = 10 mV, Vdc = open-circuit potential, frequency = 10,000–1 Hz, N = 41 points/decade.

### Stimulating α-Syn Aggregation

Samples supplemented with 50 mM sodium nitrate (Sigma), 50 mM sodium nitrite (Fisher Scientific), 500 μM ferrous iron chloride (Oakwood), 500 μM dopamine HCl (Alfa Aesar), 500 μM dihydrocaffeic acid (Sigma), 500 μM caffeic acid (TCI America), and 20 μM recombinant human α-syn monomer (Abcam, ab51189) were prepared. Materials were equilibrated to anaerobic conditions before use. Samples were allowed to incubate for 4 h at 37 °C under anoxic conditions.

### Dopamine Oxidation using Tyrosinase

Lyophilized *Aspergillus* tyrosinase (Worthington Biochemical) was resuspended in 50 mM phosphate buffer with 15% glycerol (pH 6.5) to create a 24 kU/mL solution (50 mg/mL, 390 μM). Next, 250 U (3.9 μM) tyrosinase was added to a 500 μM dopamine solution. The sample was incubated for 1 h at room temperature to afford a positive control of dopamine oxidation.

### Activating PVDF Membranes

Methanol-activated poly(vinylidene fluoride) (PVDF) membranes (Millipore) were used to evaluate aggregation of α-syn. Membranes were handled only by their edges using forceps cleaned with methanol. Membranes were activated by immersing them in 100% methanol (Fisher Scientific) for 1 min, in DDI water for 30 s, and finally in Tris-buffered saline (TBS) buffer for 2 min. TBS buffer was prepared with 50 mM Tris-HCl, pH 7.5 (Fisher Scientific), and 150 mM NaCl (Sigma-Aldrich). The pH was adjusted to 7.4, and then the filter was sterilized. All materials were allowed to equilibrate under anaerobic conditions before use.

### Nitroblue Tetrazolium Assay for the Detection of Quinones

The presence of quinones was detected as previously described.^11^ Under anoxic conditions, 2 μL of each sample was spotted onto a methanol-activated PVDF membrane. Additionally, 2 μL of dopamine (500 μM) was spotted onto the membrane as a negative control, while 2 μL of dopamine (500 μM) oxidized by *Aspergillus* tyrosinase was spotted as a positive control. After the membrane was dried for 1 h, it was reactivated before incubating with 0.6 mg/mL nitroblue tetrazolium (Santa Cruz Biotechnology) in potassium glycinate buffer, pH 10, for 45 min in the dark. The membrane was washed twice with 0.16 M sodium borate (Sigma) and left to incubate overnight. DDI water was used to wash the membrane twice before visualizing.

### Immunodetection of α-Syn Aggregates

Under anoxic conditions, 2 μL of each sample was spotted onto the methanol-activated PVDF membranes. As controls, 2 μL (578 ng) of monomeric α-syn (Abcam, ab51189) and 2 μL (578 ng) of aggregated α-syn (Abcam, ab218817) were spotted onto each membrane. The membranes were allowed to dry for 1 h. Two of the membranes were then blocked for 1 h using 5% Blotting grade Blocker Non-Fat Dry Milk (Bio-Rad) in TBS (prepared as previously described). One of the blocked membranes was then probed with the MJFR-14 antiaggregate α-syn primary antibody (Abcam, ab209538; 1:10,000 dilution) for 2 h. After four 5 min washes with TBS-T (0.1% Tween 20 (Sigma-Aldrich) in the prepared TBS), the membrane was subsequently immunostained with IRDye 800CW donkey antirabbit IgG secondary antibody (LI-COR, 926–32213; 1:20,000 dilution) for 45 min in the dark. Four more washes using TBS-T followed, before removing Tween 20 by rinsing with TBS. The other blocked membrane was immunostained with the 5G4 antiaggregate primary antibody (Millipore Sigma, MABN389; 1:4000 dilution) and IRDye 800CW donkey antimouse IgG secondary antibody (LI-COR, 926–32212; 1:20,000 dilution). The same procedure as that described above was followed to prepare the membranes. The membranes were visualized by using an Odyssey CLx imager (LI-COR) on the 800 nm channel. Blots were quantified using Image Studio data analysis software (LI-COR) to analyze the relative aggregation of α-syn. Prism was used for further data processing.

### Ponceau Staining

The remaining membrane was used as a quantitative control to ensure that the protein concentration in each sample was equivalent. The dried membrane was washed with DDI water before being submerged in Ponceau stain, which had been prepared to contain Ponceau S (Sigma-Aldrich) and glacial acetic acid (Fisher Scientific). Ponceau stain was discarded after 2 min, and the membrane was subsequently washed with DDI water to remove excess sulfide before visualizing.

### General Cell Culture Methods

Enteroendocrine STC-1 cell line (CRL-3254) was purchased from the American Type Culture Collection (ATCC). Cells were cultured in Dulbecco’s Modified Eagle Medium (DMEM, Corning) containing 4.5 g/L glucose, 2 mM l-glutamine, 10% (v/v) fetal bovine serum (Life Technologies), penicillin (100 U/mL), and streptomycin (100 μg/mL, Gibco). Cells were incubated at 37 °C in a humidified atmosphere containing 5% CO_2_. Cells were serially passaged using 0.25% Trypsin-EDTA (Gibco).

### Nitrite, Nitrate, and Inhibitor Incubation

STC-1 cells were seeded onto two μ-slide 8-well high glass bottom chamber slides (ibidi) at a density of 1 × 10^5^ viable cells/well. Cells were counted using a Countess II instrument (Invitrogen). After incubating for 48 h, the growth medium was replaced. Fresh media were supplemented with 50 mM sodium nitrate or 50 mM sodium nitrite and with 25, 50, or 100 μM dihydrocaffeic acid, caffeic acid, or vehicle. Cells were then incubated for another 24 h before fixation.

### Immunofluorescence Staining

All supplemented media were aspirated. To fix the cells, 10% formalin (Fisher Scientific) was used for 20 min. The fixative solution was then discarded, and the cells were washed twice for 5 min using sterile-filtered phosphate-buffered saline (PBS, pH 7.4). All wash steps used PBS for 5 min each. Cells were then permeabilized using 0.1% Triton X-100 (Bio-Rad, 1610407) in PBS for 20 min. After the permeabilization solution was discarded, the samples were washed twice. The cells were then blocked for 1 h using 5% normal goat serum (Thermo Scientific) and 0.2% bovine serum albumin (BSA; Fisher Scientific) in PBS. The samples were washed three times. Samples were then incubated in PBS containing 0.2% BSA with either MJFR-14 antiaggregate primary antibody (Abcam, ab209538; 1:250 dilution), isotype control (Abcam, ab172730; 1:566 dilution), or vehicle overnight at 4 °C. After washing three times, all chambers were incubated with the antigoat Alexa Fluor-488 secondary antibody (Abcam, ab150077; 1:500 dilution) for 1 h in the dark. All subsequent steps were performed in the dark. After washing three times, ∼10 drops of VECTASHEILD PLUS Antifade Mounting Medium with DAPI (Vector Laboratories) were added to each well. All demarcation and staining steps were performed at room temperature, but the chamber slides were stored at 4 °C in the dark.

### Structured Illumination Microscopy

A Zeiss Elyra 7 super-resolution microscope with a 63× oil immersion lens was used to image the cells. Three images for each sample were taken. Images were collected using 405 and 488 nm laser lines for excitation. The emission filters used were BP 420–480 and BP 495–550. Z-stack images were obtained and processed using SIM2 scaled to the raw image. Quantification of the fluorescence signal for each sample was determined by obtaining the mean intensity of the maximum intensity projection for each image given by Zen Black 3.0 software. The number of cells in each image was counted, and the mean intensity per cell was calculated. The background fluorescence signal was accounted for each replicate by subtracting the mean intensity per cell for the respective untreated sample. Results are expressed as arbitrary units (au) of mean intensity per cell. Data were plotted using Prism.

## Supporting Information Available

Effect of the Ag deposition method on the stability of the reference electrode in the printed sensors (Figure S1); photos of the anaerobic chamber used in experiments with sensors (Figure S2); the SWV of three individual sensors (Figure S3); stability of the sensor after an extended duration in high DA concentrations (Figure S4); impedance measurements for α-syn aggregation standards (Figure S5); representative membrane spotted with samples containing EDTA and ATP immunostained with clone 5G4 and its corresponding quantification (Figure S6); representative images of STC-1s incubated with DHCA or CA at lower concentrations (25 and 50 μM) and isotype controls for experiments with STC-1s (Figure S7) (PDF)
