# Supplementary material for: Mechanistic Insight into Intestinal α-Synuclein Aggregation in Parkinson’s Disease Using a Laser-Printed Electrochemical Sensor
Source: ACS Chem Neurosci. 2024 Jul 3;15(14):2623–32. doi: 10.1021/acschemneuro.4c00106 (PMC11258680; doi:10.1021/acschemneuro.4c00106)
Supplement: Supplementary file 1 — cn4c00106_si_001.pdf [file cn4c00106_si_001.pdf]

Supplementary Information for:

Mechanistic Insight into Intestinal α-Synuclein Aggregation in Parkinson's Disease using a Laser-Printed Electrochemical Sensor

Julia M. Balsamo[1†], Keren Zhou[2,3†], Vinay Kammarchedu[2,3], Aida Ebrahimi[2,3,4*], Elizabeth N. Bess[1,5*]

[1]Department of Chemistry, University of California, Irvine, CA 92617, USA

[2]School of Electrical Engineering and Computer Science, The Pennsylvania State University, University Park, PA 16802, USA

[3]Materials Research Institute, The Pennsylvania State University, University Park, PA 16802, USA

[4]Department of Biomedical Engineering, The Pennsylvania State University, University Park, PA 16802, USA

[5]Department of Molecular Biology and Biochemistry, University of California, Irvine, CA 92617, USA

†J.M.B and K.Z. contributed equally to this work

*Corresponding authors: Aida Ebrahimi, Department of Electrical Engineering, The Pennsylvania State University, University Park, PA 16802, email: sue66@psu.edu;

Elizabeth N. Bess, Departments of Chemistry and Molecular Biology & Biochemistry, University of California, Irvine, 1102 Natural Sciences II, Irvine, CA 92617, email: elizabeth.bess@uci.edu

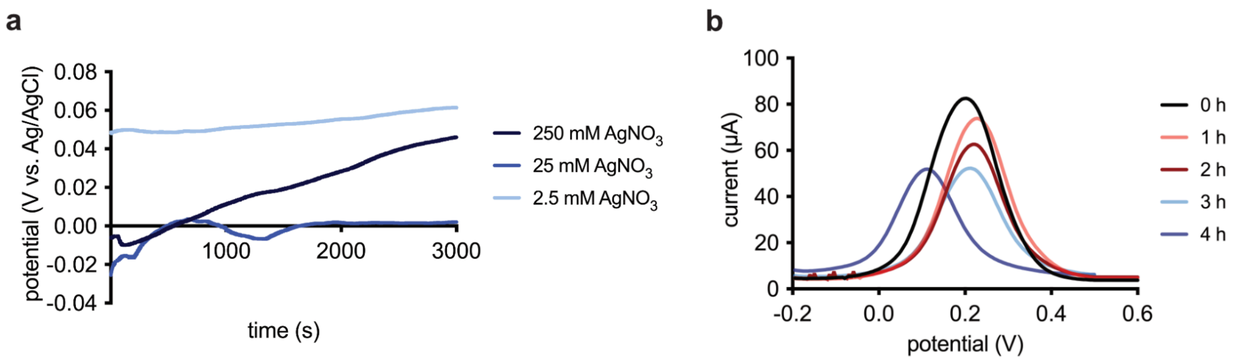

Figure S1. Effect of the Ag deposition method on the stability of the reference electrode in the printed sensors. **(a)** The electrode potential of the pRE prepared by direct laser writing with $AgNO_3$ (2.5, 25, and 250 mM concentrations) v.s. Ag/AgCl RE. **(b)** The SWV of DA (500 uM) measured at 1-hour intervals over 4 hours using the pRE prepared using direct laser writing.

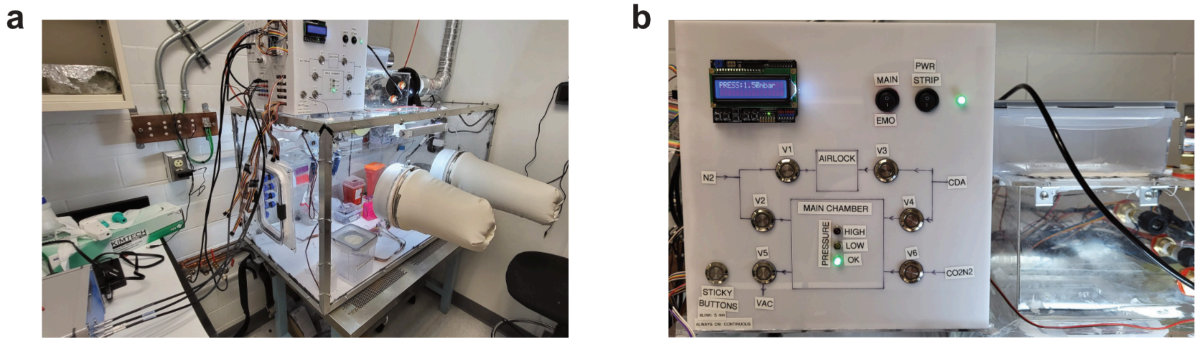

Figure S2. Photos of the anaerobic chamber used in experiments with sensors, featuring **(a)** an overview of the chamber and **(b)** the control panel.

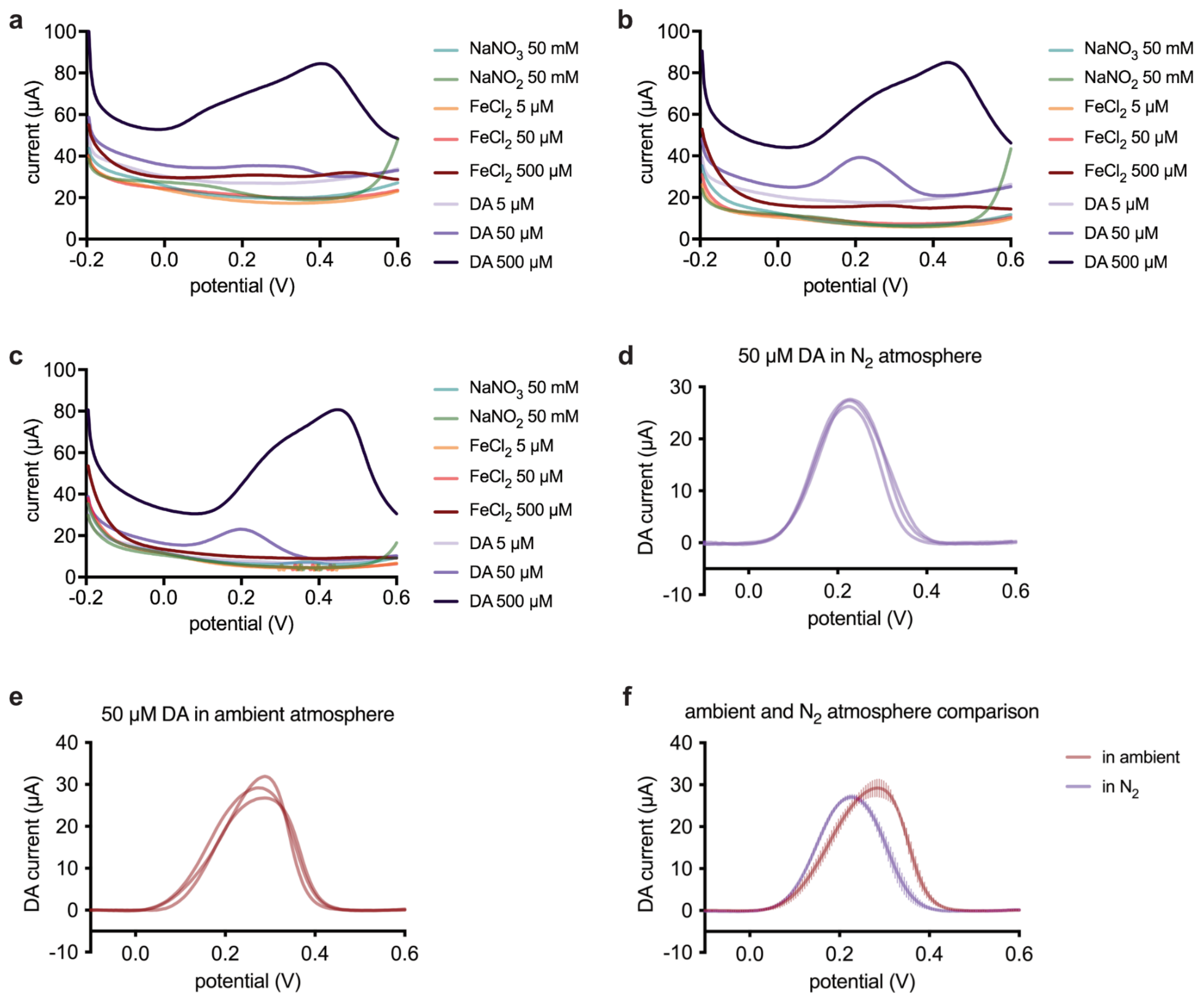

Figure S3. The SWV of 3 individual sensors **(a–c)** composed of electrodeposited Ag pRE with $NaNO_3$, $NaNO_2$, DA, and $FeCl_2$ at the concentrations noted, measured in the glovebox purged with 20% CO_2 and 80% N_2 mixture. **(d)** The baseline-subtracted SWV of the 3 individual sensors for 50 μM DA in the N_2 glovebox and **(e)** ambient atmosphere. **(f)** Overlay of **d** and **e** (all error bars represent S.E.M. for n = 3 individual sensors).

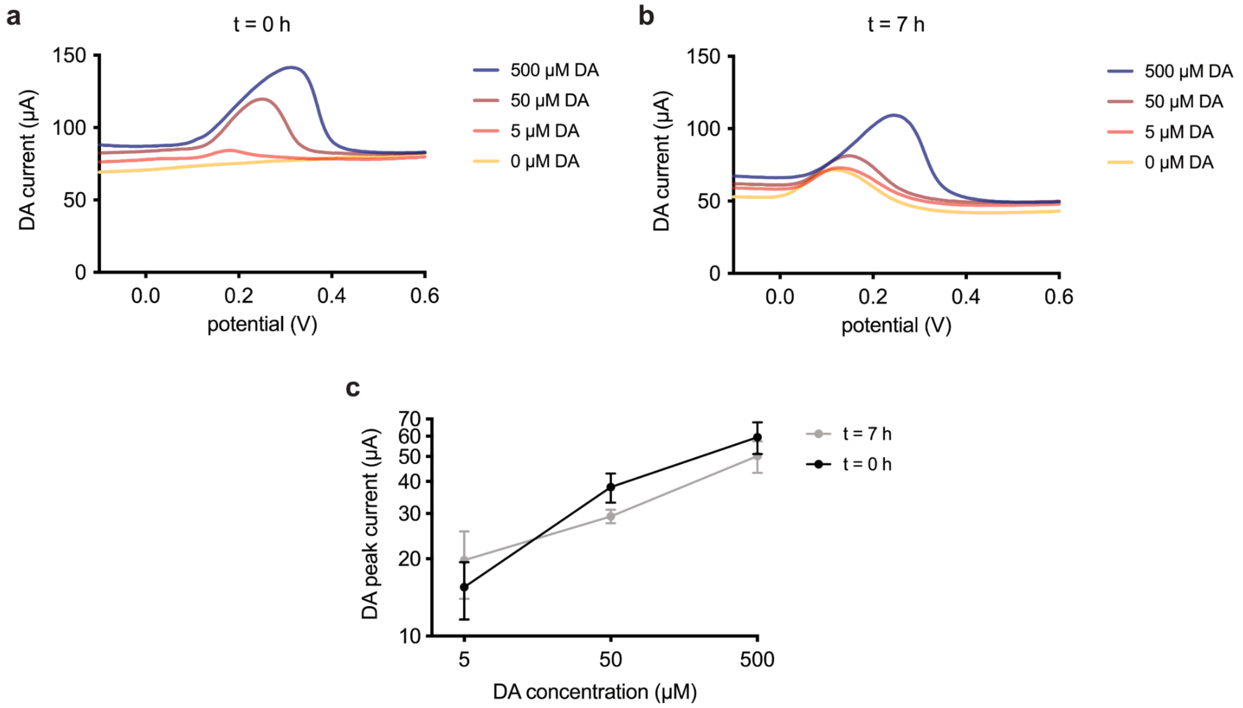

Figure S4. Representative SWV curves at various DA concentrations (0, 5, 50, and 500 μM) **(a)** before and **(b)** after immersion in 500 μM DA for 7 h. **(c)** Calibration curves based on the SWV DA peak current data at 0 h and 7 h.

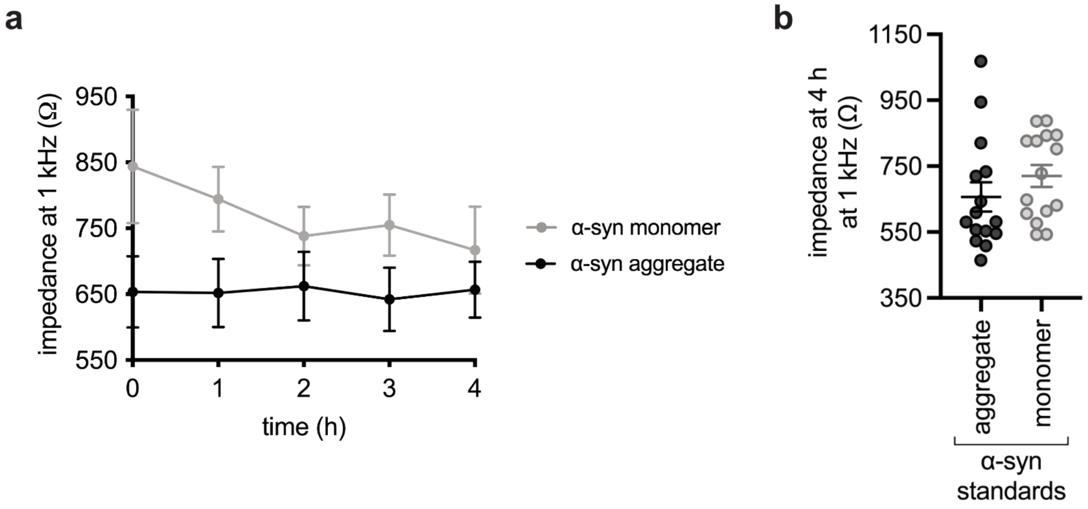

Figure S5. Impedance measurements for α-syn aggregation standards. **(a)** Impedance measurements at 1 kHz for α-syn monomer and commercial α-syn aggregate over 4 h. **(b)** Impedance at 4 h for each standard.

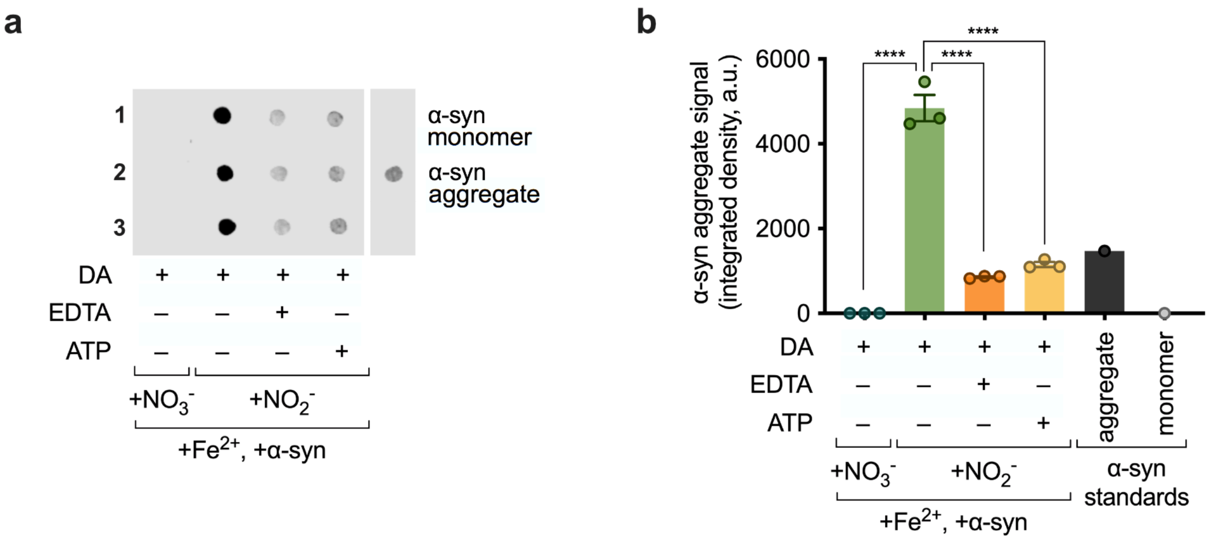

Figure S6. Dot blots used to verify the extent of α-syn aggregation after a 4 h incubation period with or without EDTA and ATP: **(a)** Dot blot spotted with three biological replicates and immunostained with anti-aggregate primary antibody 5G4. **(b)** End-point quantification of α-syn aggregation in **a** (all error bars represent S.E.M. for n = 3 samples; significance determined by one-way ANOVA with Sidak's multiple comparisons test, ****: $P < 0.0001$).

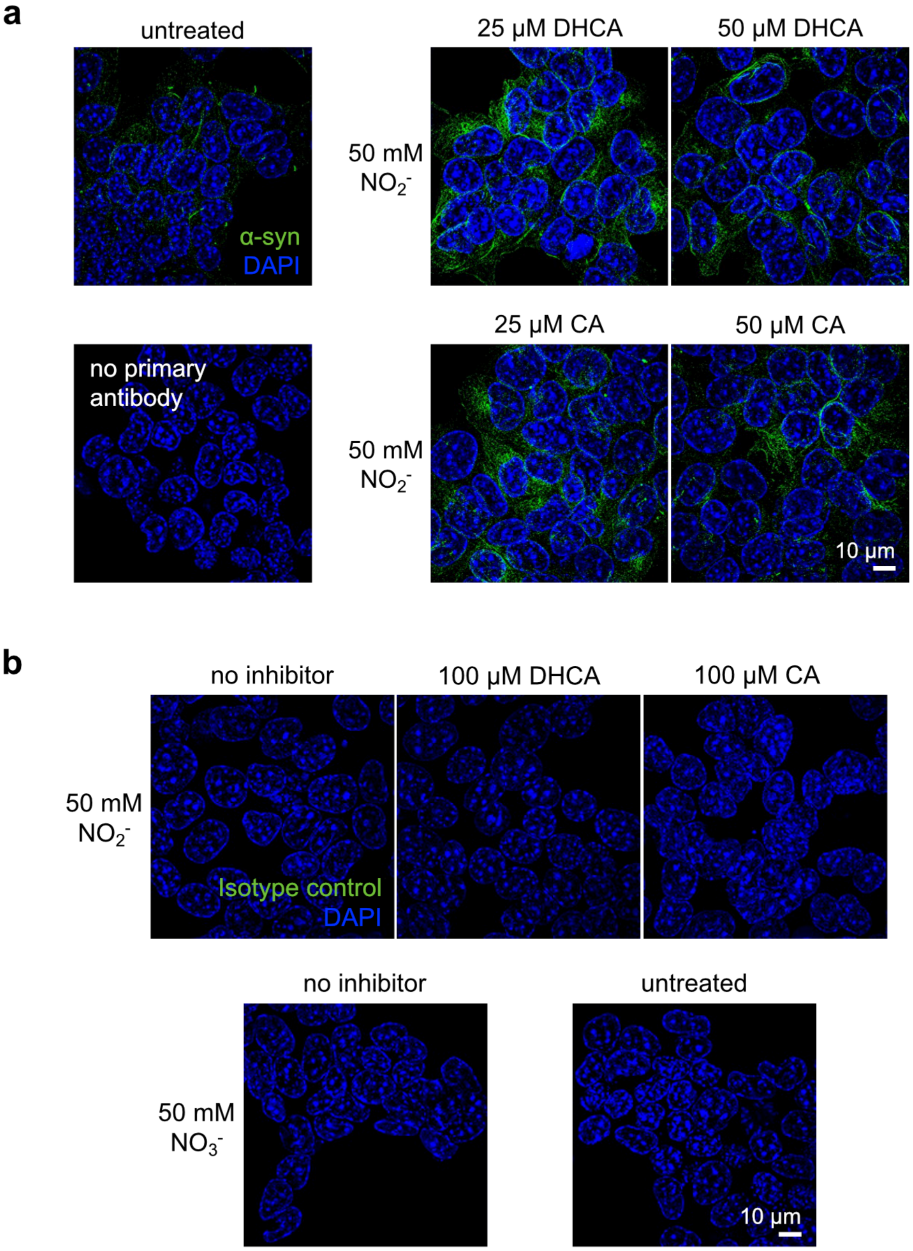

Figure S7. Induced aggregation of α-syn in gut epithelial cells is reduced in the presence of diet-derived catechols DHCA and CA. Representative images of fixed STC-1 cells incubated for 24 h with NO_2^- or NO_3^- and DHCA or CA probed with **(a)** anti-aggregate antibody MJFR-14 (α-syn aggregate signal is in green and DAPI-stained nuclei are in blue) or **(b)** isotype control.
